# Cardiovascular aging: the mitochondrial influence

**DOI:** 10.20517/jca.2023.22

**Published:** 2023-07-17

**Authors:** Shakti Sagar, Asa B. Gustafsson

**Affiliations:** Skaggs School of Pharmacy and Pharmaceutical Sciences, University of California San Diego, La Jolla, CA 92093, USA

**Keywords:** Aging, mitochondria, heart disease

## Abstract

Age-associated cardiovascular disease is becoming progressively prevalent due to the increased lifespan of the population. However, the fundamental mechanisms underlying the aging process and the corresponding decline in tissue functions are still poorly understood. The heart has a very high energy demand and the cellular energy needed to sustain contraction is primarily generated by mitochondrial oxidative phosphorylation. Mitochondria are also involved in supporting various metabolic processes, as well as activation of the innate immune response and cell death pathways. Given the central role of mitochondria in energy metabolism and cell survival, the heart is highly susceptible to the effects of mitochondrial dysfunction. These key organelles have been implicated as underlying drivers of cardiac aging. Here, we review the evidence demonstrating the mitochondrial contribution to the cardiac aging process and disease susceptibility. We also discuss the potential mechanisms responsible for the age-related decline in mitochondrial function.

## INTRODUCTION

Improved health care, hygiene, diet, healthier lifestyles, and medical care have all contributed to a substantial increase in lifespan, with an average life expectancy of 76.1 years in the US [Life Expectancy in the USA. Dropped for the Second Year in a Row in 2021 (cdc.gov)]. Unfortunately, this has also led to an increase in many age-associated diseases such as dementia, cancer, and cardiovascular disease (CVD). In fact, aging alone represents one of the biggest risk factors for developing CVD. A better understanding of the mechanisms underlying the aging process is urgently needed so that improved therapies can be developed that prevent or treat age-related diseases.

The beating heart is a highly energy-consuming organ and the cellular energy needed to sustain contraction is primarily generated by mitochondrial oxidative phosphorylation (OXPHOS). Mitochondria are also involved in supporting various metabolic processes, as well as activation of the innate immune response and cell death pathways^[1]^. Thus, the heart is highly susceptible to the effects of mitochondrial dysfunction. Mitochondria have been directly implicated as underlying drivers of cardiac aging. Studies have reported that the age-related decline in cardiac function is partly attributed to dysregulation of mitochondrial function and a decline in mitochondrial quality control. The aged heart accumulates dysfunctional mitochondria that are deficient in ATP generation and become major sources of reactive oxygen species (ROS) and oxidative stress^[2]^. Interestingly, various interventions or treatments that directly or indirectly target mitochondria to reduce ROS generation, promote oxidative metabolism or enhance quality control have all been demonstrated to delay cardiac aging and alleviate disease development^[3–7]^.

Aging is a complex biological process that is associated with a gradual decline in the function of various tissues, leading to increased susceptibility to stress and disease development. However, the fundamental mechanisms contributing to the aging process and development of age-related pathologies are still poorly understood. Given the central role of mitochondria in energy metabolism and cell survival, unraveling the mechanisms underlying age-related mitochondrial impairments is currently of great interest. In this review, we describe existing evidence implicating mitochondrial impairment in the cardiac aging process and disease susceptibility. We also discuss the potential mechanisms responsible for the age-related decline in mitochondrial function and how alterations in the signaling pathways regulating mitochondrial quality control contribute to the accumulation of dysfunctional mitochondria.

## MITOCHONDRIAL DYSFUNCTION IN THE AGING HEART

### Oxidative stress

**Figure 1.**
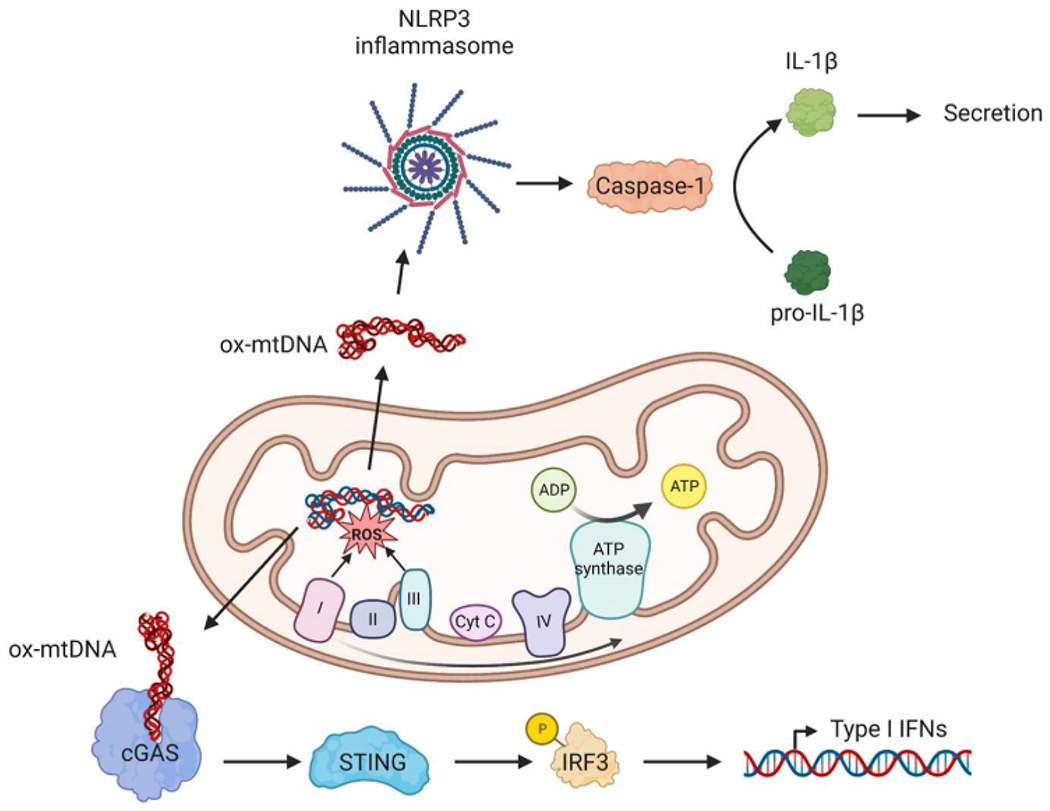
Mitochondrial dysfunction leads to activation of inflammation. Release of oxidized mitochondrial DNA (ox-mtDNA) can induce pro-inflammatory signaling pathways via activation of the NLRP3 inflammasome or cGAS-STING pathways. Mt-DNA dependent NLRP3 activation leads to caspase-1 activation and maturation of proinflammatory cytokines such as IL1β. cGAS recognizes mtDNA in the cytosol and activates STING. STING phosphorylates IRF3 which induces a type I interferon transcriptional response (Created with BioRender.com).

Mitochondria are the main source of ROS in the aging heart. They are generated as a by-product of electron transfer during oxidative phosphorylation (OXPHOS) when electrons that leak out of the electron transfer chain react with oxygen to produce superoxide. The superoxide can be converted to hydrogen peroxide by superoxide dismutase 2 in the matrix. The hydrogen peroxide, in turn, can be converted into a highly reactive hydroxyl radical. Complex I and III of the respiratory chain are the main sites of ROS production in mitochondria [Figure 1]. NAD(P)H oxidase, xanthine oxidase, and nitric oxide synthase are additional sources of ROS in the heart^[8]^.

Because of their high reactivity with lipids, proteins, and DNA, ROS pose a constant threat to mitochondrial function. To ensure a reliable supply of energy, cardiac myocytes are equipped with a strong antioxidant system that quickly neutralizes the various ROS species and minimizes oxidative damage. However, excessive ROS generation by dysfunctional mitochondria can lead to an imbalance between production and removal, resulting in oxidative stress^[9]^. Many genes involved in ROS homeostasis are downregulated in cardiac tissues of elderly humans and rats, which correlate with increased superoxide production^[10]^. A key role for mitochondrial ROS in cardiac aging and lifespan has been demonstrated in studies utilizing mice with overexpression of a mitochondria-targeted catalase (mCAT). Catalase mitigates the toxic effects of hydrogen peroxide by catalyzing its conversion to water and oxygen and targeting this enzyme to mitochondria attenuates cardiac aging and prolongs median lifespan in mice^[11,12]^. Hearts of aged mCAT mice also have diminished characteristics of aging, including reduced mitochondrial protein oxidation and mtDNA damage, as well as decreased fibrosis and fiber size. In addition, reducing oxidative stress pharmacologically in aged mice through infusion of a mitochondria-targeted antioxidant, MitoTEMPO, improves cardiac function^[4]^. Similarly, delivery of SS-31 (also known as elamipretide), a synthetic tetrapeptide that associates with the mitochondrial phospholipid cardiolipin, for 8 weeks improves diastolic function and reduces oxidative stress in the hearts of aged mice^[7]^. Overall, these studies suggest that ROS generated by mitochondria contribute to the age-related decline in cardiac function and their targeting represents a potential approach to preserve the health of the aging heart.

### Mitochondrial DNA damage

Although mitochondria have transferred most of their genome to the nucleus, they still contain their own DNA which encodes tRNAs, rRNAs, and thirteen critical OXPHOS subunits necessary for mitochondrial function. Due to the location near the OXPHOS complexes and the lack of protective histones, mtDNA is vulnerable to oxidative damage^[9]^ [Figure 1]. MtDNA mutations, duplications, and deletions are known to accumulate over time in various tissues and are believed to directly contribute to the aging process and disease development. For example, Tranah *et al*. reported a correlation between the accumulation of damaged mtDNA in elderly individuals and decreased strength, cognition, metabolism, and cardiovascular health^[13]^. They further noted that individuals with a higher burden of mtDNA mutations are at an increased risk of dementia and stroke. In addition, a specific 4977 bp mtDNA deletion that results in the loss of several genes encoding important OXPHOS subunits is commonly observed in cells from aged tissue^[14]^. This deletion is associated with various age-related diseases, including Alzheimer’s disease and CVD^[15–17]^.

Studies in the “mtDNA mutator” mice have provided direct evidence that mtDNA mutations can contribute to the aging process. These mice have a knock-in mutation at D257A in the mitochondrial DNA Polymerase gamma (POLG) that causes inactivation of the proofreading exonuclease activity^[18,19]^. The POLG mutant mice have an increased burden of somatic mtDNA mutations and develop premature aging with thymic involution, osteoporosis, anemia, alopecia, kyphosis, and reduced lifespan. The accumulation of mtDNA also leads to increased nuclear DNA strand breakage and oxidative damage^[20]^. These mice also develop cardiac hypertrophy at a younger age than age-matched WT mice^[18]^. Overall, studies in these mice clearly demonstrate a relationship between mtDNA mutations and aging. However, it is important to bear in mind that although the POLG mutant mice are suitable for examining the consequences of mtDNA mutations, the mutational load in these mice is significantly higher than is found in normal aging and findings might not reflect what occurs in normal physiological aging.

Increased oxidative stress within the mitochondria is thought to play a significant role in the aging process^[9]^. ROS produced inside mitochondria cause mtDNA damage which affects the synthesis of functional respiratory chain subunits. This, in turn, can impact mitochondrial respiration and further increase ROS production and mtDNA damage. As discussed in the next section, oxidized mtDNA is also a potent activator of the innate immune system, leading to chronic inflammation. Thus, the continuous ROS-mtDNA damage cycle likely underlies the aging process and onset of age-associated diseases^[9]^. Several studies have linked oxidative stress to tissue dysfunction and the premature aging phenotype in POLG mutator mice^[21–24]^. In addition, overexpression of mCAT in POLG mutant mice partially rescues the age-dependent cardiomyopathy that these mice develop at 13-14 months of age. Suppression of mitochondrial hydrogen peroxide levels in these mice leads to reduced cardiac hypertrophy and dilatation, improved systolic and diastolic function, and decreased cardiac fibrosis^[5]^. Also, a recent study reported that increased oxidative stress and chronic inflammation are responsible for the age-dependent decline in cardiac function in POLG mutant mice^[25]^. Overall, these findings clearly demonstrate that mtDNA mutations and oxidative stress can be driving forces of aging.

### Mitochondria and inflammation

Inflammation is a response aimed at limiting and repairing damage caused by acute traumatic injury or invading pathogens. However, chronic low-grade inflammation is another major contributor to various age-related pathologies and biological aging. Dysfunctional mitochondria are thought to underlie the chronic inflammation in tissues through activation of the innate immune response. Because of their bacterial origin, mitochondrial DNA (mtDNA) is a potent damage-associated molecular pattern (DAMP) and induces an inflammatory response by activating pathways that are normally involved in pathogen-associated responses^[26]^. For instance, oxidized fragmented mtDNA in the cytosol activates the NLR family pyrin domain containing 3 protein (NLRP3) inflammasome^[27]^ [Figure 1]. The NLRP3 inflammasome activates caspase-1 which initiates the processing and secretion of pro-inflammatory cytokines IL-1β and IL-18. The NLRP3 inflammasome has been extensively studied in cardiovascular diseases and many studies have demonstrated that its activation promotes cardiac inflammation, pathological remodeling and heart failure development^[27]^. NLRP3 inflammasome activation is also an underlying factor in low-grade age-related inflammation. NLRP3-deficient mice have improved health spans with attenuated age-related changes, including reduced bone loss, improved cognitive function, and motor performance compared to aged WT mice^[28]^. Furthermore, NLRP3^−/−^ mice have increased muscle strength and endurance compared to age-matched WT mice^[29]^, suggesting that these mice are protected from the loss of muscle mass that occurs with age. In contrast to WT mice, NLRP3-deficient mice do not develop cardiac hypertrophy and fibrosis with age and have extended lifespans^[30]^. Overall, these findings suggest that cardiac inflammation due to mitochondrial dysfunction and NLRP3 activation contributes to age-related inflammation and that limiting activation of the inflammasome downstream of mitochondria can limit cardiac aging.

Moreover, cytoplasmic mtDNA can also activate the innate immune system via the cyclic GMP-AMP synthase (cGAS)-stimulator of interferon genes (STING) signaling pathway, a major driver of type I interferon-mediated inflammatory responses^[31,32]^. The cGAS protein functions as a cytoplasmic mtDNA sensor and activates STING, which in turn phosphorylates the transcription factor IRF3, leading to the expression of type I IFN genes [Figure 1]. Activation of the cGAS-STING pathway has been linked to age-related neurodegeneration^[33]^ and cellular senescence^[34]^. Accumulation of mtDNA mutation POLG mutator mice also leads to aberrant activation of the cGAS-STING signaling pathway in macrophages which contributes to the cardiac dilatation and increased mortality in these mice^[25]^. These mice have persistent activation of the cGAS-STING–IFN-I axis in macrophages and tissues, which leads to repression of nuclear factor erythroid 2-related factor 2 (NRF2) activity. NRF2 is a transcription factor that is involved in the cell’s antioxidant defense by inducing transcription of antioxidant and cytoprotective genes^[35]^. Disrupting the IFN-I signaling in POLG mutator mice delays the age-dependent decline in cardiac function and extends lifespan^[25]^, confirming the contribution of persistent inflammation to the accelerated aging phenotype in these mice. Thus, these studies clearly demonstrate a link between mitochondria and the innate immune response in aging.

### Mitochondrial protein quality control

**Figure 2.**
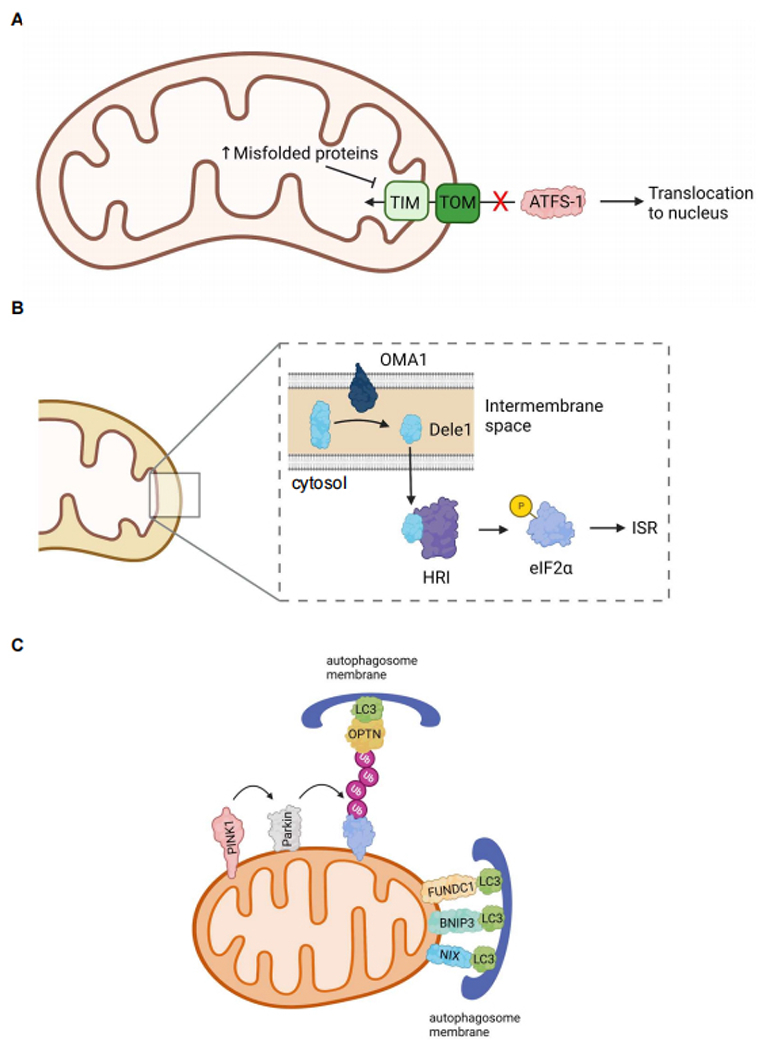
Mitochondrial quality control pathways. (A) Activation of the *UPR^mt^* in *C.elegans*. Mitochondrial stress leads to inhibition of TOM/TIM-mediated mitochondrial import of ATFS-1. This allows ATFS-1 to re-localize to the nucleus where it proceeds to activate a transcriptional response aimed at restoring mitochondrial homeostasis. (B) Activation of the integrated stress response (ISR) in mammalian cells. The protease OMA1 cleaves Dele1 in response to mitochondrial stress. Dele1 activates HRI in the cytosol which phosphorylates eIF2a leading to activation of the ISR. (C) Regulators of mitochondrial autophagy. PINK1 accumulates on the outer membrane of dysfunctional mitochondria where it recruits the E3 ubiquitin ligase Parkin. Parkin-mediated ubiquitination leads to binding of adaptor proteins such as OPTN and LC3 on the growing autophagosome membrane. Mitophagy receptors in the outer mitochondrial membrane facilitate ubiquitin independent autophagic sequestration of mitochondria (Created with BioRender.com).

Mitochondrial protein homeostasis is ensured by an intricate protein quality-control network composed of resident chaperones and proteases that are regulated by the mitochondrial unfolded protein response (*UPR^mt^*)^[1]^. Because mtDNA does not encode any stress response genes, mitochondria must communicate the stress to the nucleus to launch a repair response^[36]^. The *UPR^mt^* is a conserved specific transcriptional stress response program aimed at reducing misfolded proteins inside mitochondria. Studies in worms have provided important insights into this stress response and ATFS-1 has been identified as a key regulator of the *UPR^mt^* in *C.elegans* [Figure 2A]. This transcription factor contains both a mitochondrial targeting sequence (MTS) and a nuclear localization sequence (NLS). Under normal conditions, ATFS-1 is actively imported into mitochondria, where it is degraded by the Lon Protease in the matrix^[37]^. However, upon mitochondrial stress, the import of ATFS-1 into the matrix is abrogated, allowing it to re-localize to the nucleus and activate transcription of mitochondrial chaperones and other genes required for repair^[38–40]^. ATF5 has been reported to function as a mammalian orthologue of ATFS-1^[37]^. Like ATFS-1, ATF5 induces transcription of mitochondrial chaperones and proteases to reduce proteotoxic stress. ATF5 overexpression also restores the *UPR^mt^* response in ATFS-1-deficient worms^[37]^, further confirming its function in communicating mitochondrial stress to the nucleus.

The activity of the *UPR^mt^* pathway influences the longevity of organisms and its induction correlates with increased lifespan. For example, experiments conducted in *C. elegans* demonstrate that expression of mitochondrial chaperones and proteases gradually increases with age and that worms with increased *UPR^mt^* activation at baseline have longer lifespans^[41,42]^. Moreover, Snell dwarf mice have prolonged life and health spans, and analysis of cultured cells from these mice revealed increased levels of two *UPR^mt^* genes, the mitochondrial chaperone HSP60 and mitochondrial protease LONP1^[43]^. Because a decline in protein quality control leads to the accumulation of damaged and nonfunctional proteins, it is not unexpected that maintaining or enhancing the *UPR^mt^* can preserve mitochondrial function in aging cells. However, studies specifically aimed at *UPR^mt^* in aged hearts are currently lacking.

### Mitochondrial activation of the integrated stress response

Mitochondrial stress also activates the integrated stress response (ISR) via phosphorylation of eukaryotic translation initiation factor 2 alpha (eIF2α), which leads to decreased global protein synthesis and induction of specific genes such as ATF4 (activating transcription factor 4)^[44]^. It is well established that the ISR protects hearts against stress and disease development^[45]^ and recent studies also suggest that the ISR is involved in suppressing aging. For example, overexpression of ATF4 increases lifespan in *C. elegans*^[46]^ and elevated ATF4 activity correlates with increased lifespan in various mouse models^[47]^. In the heart, ATF4 provides protection by regulating transcription of genes involved in the antioxidative response and NADPH production^[48]^. A recent study discovered that ATF4-mediated longevity in *C. elegans* involves induction of various small heat shock proteins as well as induction of cystathionine-γ lyase-2 (CTH-2), an enzyme that catalyzes the formation of hydrogen sulfide (H2S)^[46]^. Conversely, loss of Atg4 in hematopoietic stem cells leads to defects associated with an aging-like phenotype^[49]^.

A novel pathway of ISR activation involving mitochondria was recently identified where dysfunctional mitochondria activate the ISR via DAP3 Binding Cell Death Enhancer 1 (Dele1)^[50,51]^ [Figure 2B]. Upon mitochondrial dysfunction, Dele1 is cleaved by Oma1, a mitochondrial protease in the inner membrane^[50,51]^. The cleaved Dele1 fragment activates the eIF2a kinase heme-regulated inhibitory (HRI) in the cytosol. HRI, in turn, phosphorylates eIF2α to turn on the ISR^[50,51]^. The importance of Dele1 in communicating mitochondrial stress and activating the ISR in the heart was recently demonstrated by two different groups. Ahola *et al*. reported that ISR signaling via Oma1-Dele1-Atf4 protects hearts against the development of cardiomyopathy in mice with OXPHOS deficiency^[52]^. This group also demonstrated that cardiac-specific deletion of *Dele1* leads to accelerated disease development in the OXPHOS mutant mice. Similarly, Huynh *et al*. demonstrated that deletion of Dele1 in myocytes is detrimental in mouse models of fetal and adult mitochondrial cardiomyopathy^[53]^. Interestingly, this study monitored mice with cardiac-specific *Dele1*-deficiency for up to one year and noted that the lack of Dele1 in myocytes has no effect on cardiac structure or function at baseline. The lack of an accelerated aging cardiac phenotype suggests that Dele1 is not critical for baseline homeostasis and suppression of normal aging. Instead, it is likely that the primary function of this pathway is in the adaptation to severe stress. Since aged individuals are known to be more susceptible to stress, it will be interesting to investigate if activation of this pathway is decreased with age.

### Autophagy and mitophagy

When damage to a mitochondrion exceeds the repair capacity, the entire organelle must be eliminated before it causes harm to the cell. The primary mechanism by which mitochondria are removed occurs through mitochondrial autophagy or mitophagy. In mitophagy, mitochondria are selectively engulfed by autophagosomes for delivery to lysosomes, where they are degraded^[54]^. Thus, a decline in this process is associated with the accumulation of autophagic cargo, including dysfunctional mitochondria. Mitophagy is a highly regulated process and requires coordinating induction of autophagy with labeling of mitochondria for selective autophagic degradation. Autophagosome formation is initiated upon activation of the serine/threonine kinase Ulk1^[55]^. Ulk1 activates the Beclin1-Vps34-Vps15 complex, which, in turn, recruits various autophagy-related genes (Atg) proteins responsible for autophagosome nucleation and elongation^[55,56]^. In addition, loss-of-function mutations in Atg1 (Unc-51), Atg7, Atg18, and Beclin 1 (Bec-1) decrease the lifespan of *C. elegans*^[57]^. Autophagic activity is also reduced in aged mouse hearts^[58,59]^ and the negative impact of impaired autophagic activity on cardiac aging has been confirmed in mouse models with autophagic deficiency. For instance, cardiac-specific deletion of the core autophagy protein Atg5 abrogates autophagosome formation in mouse hearts and leads development of cardiac dysfunction and accelerated cardiac aging^[59,60]^. In contrast, mice with ubiquitous overexpression of Atg5 in tissues have an anti-aging phenotype, including extended lifespans^[61]^. In addition, interventions that enhance autophagy extend the lifespans of organisms and improve cardiovascular health. Many studies have demonstrated that longevity is linked to the activation of autophagy^[62]^. In fact, interventions, including caloric restriction and spermidine, that are known to extend lifespan in various organisms depend on autophagy to promote longevity^[63–66]^. Also, rapamycin is a potent inducer of autophagy and long-term treatment ameliorates many age-related effects, improves cardiac function and extends lifespan^[7,67]^. Although Rapamycin affects many pathways, its effect on longevity is lost when autophagy is impaired, confirming that some of its anti-aging effects depend on autophagic activity^[68,69]^. Moreover, Beclin1 is sequestered by Bcl-2 under baseline conditions, which prevents it from forming the complex with Vps34 to initiate autophagosome formation^[70]^. Mice with a knock-in mutation in *Becn1* that disrupts its interaction with Bcl-2 have increased basal autophagic activity in tissues as well as extended lifespan and health span^[71]^. At 20 months of age, these mice have reduced cardiac hypertrophy and fibrosis compared to age-matched WT mice. Whether mitochondrial fitness is also improved in these mice remains to be investigated. Beclin1 plays a specific role in mitophagy^[56]^ and future studies should investigate if targeting Beclin1 can selectively enhance mitochondrial turnover in aging myocytes.

In mitophagy, the PINK1-Parkin pathway is involved in labeling dysfunctional mitochondria for autophagic degradation [Figure 2C]. PINK1 (PTEN-induced kinase 1) becomes stabilized on the outer mitochondrial membrane (OMM) of depolarized mitochondria^[72]^ which leads to recruitment and activation of the E3 ubiquitin ligase Parkin^[73,74]^. Parkin ubiquitinates various OMM proteins which are recognized by adaptor proteins such as p62, optineurin (OPTN), NDP52, TAX1BP1, and NBR1^[75,76]^. These adaptor proteins are responsible for linking the ubiquitinated mitochondria to LC3 on the autophagic membranes via their LC3 Interacting Region (LIR) motifs^[77]^. Several studies suggest that a decline in the removal of damaged mitochondria is associated with aging. A study utilizing fluorescent mitophagy reporter mice demonstrated a decrease in mitophagy levels in the brains of aged mice compared to young mice^[78]^. Also, PINK1 deficient mice exhibit accelerated decline in mitochondrial function and accumulation of megamitochondria in the cortex^[79]^. Cardiac mitochondria in PINK1^−/−^ mice are dysfunctional by 2 months of age with concurrent development of left ventricular dysfunction and cardiac hypertrophy^[80]^. Although Parkin and PINK1 function in the same pathway, Parkin-deficient mice have a less severe cardiac phenotype, and despite the accumulation of abnormal mitochondria at ~ 6 months of age, cardiac function is still normal^[81,82]^. This suggests that additional E3 ubiquitin ligases can function downstream of PINK1 in the absence of Parkin. Moreover, enhancing the mitophagy pathway can delay cardiac aging, where cardiac-specific overexpression of Parkin improves mitochondrial quality in aging hearts^[3]^. However, Parkin overexpression in POLG mutant mice does not rescue the accelerated cardiac aging phenotype but results in cardiac fibrosis, likely due to an imbalance between ubiquitination and autophagic degradation^[83]^. Interestingly, Parkin deficiency in POLG mutator mice does not accelerate cardiac aging^[83]^, suggesting that alternative mitophagy pathways might be activated in these hearts.

Mitophagy can also occur via a receptor-mediated pathway that involves transmembrane proteins in the outer mitochondrial membrane (OMM). There are several proteins in the OMM that can function as mitophagy receptors and the best well-characterized proteins include FUNDC1^[84]^, BNIP3^[85]^ and NIX/BNIP3L^[86,87]^. They contain a LIR motif that functions to anchor the autophagosome membrane directly to the mitochondrion via binding to LC3 [Figure 2C]. In addition, the essential phospholipid cardiolipin can facilitate mitophagy in cells. Cardiolipin is localized in the inner mitochondrial membrane but relocalizes to the outer membrane when mitochondria become damaged, where it interacts with LC3 on the autophagosome membrane^[88]^. The mitophagy receptors appear to play an important role in maintaining mitochondrial homeostasis at baseline and their levels are altered with age in tissues. For instance, FUNDC1 levels gradually decrease with age in mouse coronary arteries but are restored by exercise^[89]^. Endothelial-specific deletion of FUNDC1 eliminates the protective effects of exercise training, while overexpression of FUNDC1 protects aged mice from myocardial ischemia/reperfusion injury^[89]^. FUNDC1 is important in the heart and myocyte-specific FUNDC1 deletion in mice leads to cardiac dysfunction and heart failure^[90]^. Moreover, Bnip3 have been reported to counteract mitochondrial dysfunction in the aging brain and prolong the healthy lifespan in flies^[91]^. In this study, the authors found that neuronal induction of Bnip3 prevents the accumulation of damaged mitochondria, leading to enhanced longevity of the flies. The protective effects of Bnip3 are lost in autophagy deficiency^[91]^, confirming the importance of mitophagy in mediating protection. Similarly, Bnip3 plays a key role in homeostasis of skeletal muscle and is responsible for alleviating muscle inflammation and atrophy during aging^[92]^. Irazoki *et al*. discovered that Bnip3 expression is increased in skeletal muscle during aging, which functions to protect the tissue against aging-induced inflammation^[92]^. Knockdown of Bnip3 in cultured muscle cells leads to abrogation of mitophagy, activation of NLRP3 inflammasome and increased secretion of the pro-inflammatory cytokine IL-1β. Importantly, elevated levels of Bnip3 in aged human skeletal muscle correlate with low inflammatory profile^[92]^.

Currently, studies investigating the impact of aging on mitophagy receptors in the heart are lacking. However, simultaneous loss of Bnip3 and Nix in the mouse heart leads to accelerated accumulation of dysfunctional mitochondria and cardiac dysfunction in mice^[93]^, suggesting that these proteins are important in the normal turnover of mitochondria to maintain homeostasis. Bnip3 protein levels are also increased in aged mouse hearts^[58]^, which might represent an attempt by the heart to increase turnover of less efficient old mitochondria. However, because mitophagy relies on autophagosome formation which is reduced in the aged heart^[58,59]^, increasing Bnip3 levels without restoring autophagosome formation will be ineffective.

## THERAPEUTIC TARGETING OF MITOCHONDRIA IN AGING

Currently, effective treatments to prevent age-related cardiovascular dysfunction are lacking, but there is a strong interest in developing therapeutics that are aimed at preserving or improving mitochondrial health in cells. Many interventions that protect against cardiac aging, including caloric restriction^[94]^, exercise^[95,96]^, and nicotinamide riboside^[97]^, spermidine^[98]^ or rapamycin^[67,99]^ treatments, are mediated at least in part through the preservation of mitochondria. Pre-clinical studies clearly suggest that directly targeting mitochondrial ROS production or enhancing repair and turnover may have promising therapeutic benefits in the aging heart. For example, administration of the mitochondria-targeted antioxidant MitoTEMPO to aged mice reduces oxidative stress and improves systolic and diastolic function^[4]^, while MitoQ administration ameliorates vascular endothelial dysfunction in aged mice^[6]^. Treatment of aged mice with the mitochondrially-targeted tetrapeptide SS-31 (elamipretide) for 8 weeks leads to reduced oxidative stress in hearts with improvements in cardiac function and reversal of cardiac hypertrophy^[7]^. There is also great interest in developing small molecules that can directly activate mitophagy in cells. VL-004 is a small molecule that increases mitophagy and longevity in *C.elegans* via *dct-1*, the worm homolog of mammalian mitophagy receptors BNIP3 and BNIP3L/NIX^[100]^. VL-004 failed to extend lifespan in *dct-1* mutant worms^[100]^, confirming that the effect on lifespan extension is dependent on mitophagy. Although these studies targeting mitophagy are promising, whether the beneficial effects are preserved in larger organisms remains to be investigated.

## CONCLUSION

**Figure 3.**
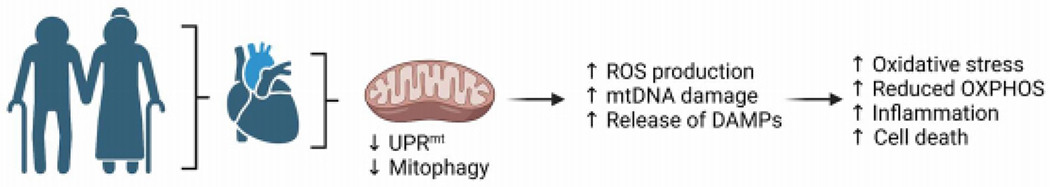
Altered mitochondrial function and turnover in the aging heart (Created with BioRender.com).

Due to the longer lifespan in the population, treatments that prevent late-life morbidities and increase health spans are urgently needed. Dysfunctional mitochondrial are clearly major contributors to cardiac aging [Figure 3]. Although rejuvenating mitochondria to reverse or prevent aging by directly targeting mitochondrial ROS or activating mitophagy seems to have anti-aging benefits, these interventions are not without risks. ROS also function as signaling molecules in cells and complete suppression of mitochondrial ROS has adverse effects on heart function^[101]^. Similarly, too much activation of mitophagy can lead to excessive clearance of mitochondria. If the clearance exceeds the cells’ capacity to generate new mitochondria, it can lead to a catastrophic energy deficiency. Thus, the therapeutic window of these interventions needs to be clearly defined. There are also alternative pathways to traditional autophagy in place that can deliver mitochondria to lysosomes^[102–105]^, but how these are affected in the aging heart has not been investigated.

In sum, mitochondria fitness is key to maintaining a healthy heart during aging, and a better understanding of the molecular basis for mitochondrial dysfunction during aging will provide increased knowledge that can be used to develop new therapies to combat age-related morbidities.

## Data Availability

Not applicable.
